# Crosstalk between Breast Milk N-Acetylneuraminic Acid and Infant Growth in a Gut Microbiota-Dependent Manner

**DOI:** 10.3390/metabo13070846

**Published:** 2023-07-13

**Authors:** Runze Ouyang, Sijia Zheng, Xiaolin Wang, Qi Li, Juan Ding, Xiao Ma, Zhihong Zhuo, Zhen Li, Qi Xin, Xin Lu, Lina Zhou, Zhigang Ren, Surong Mei, Xinyu Liu, Guowang Xu

**Affiliations:** 1 CAS Key Laboratory of Separation Science for Analytical Chemistry, Dalian Institute of Chemical Physics, Chinese Academy of Sciences, Dalian 116023, China; 2 University of Chinese Academy of Sciences, Beijing 100049, China; 3 Liaoning Province Key Laboratory of Metabolomics, Dalian 116023, China; 4 Department of Quality Control, The First Affiliated Hospital of Zhengzhou University, Zhengzhou 450052, China; 5 Department of Nursing, The First Affiliated Hospital of Zhengzhou University, Zhengzhou 450052, China; 6 Department of Pediatric, The First Affiliated Hospital of Zhengzhou University, Zhengzhou 450052, China; 7 Department of Interventional Radiology, The First Affiliated Hospital of Zhengzhou University, Zhengzhou 450052, China; 8 Academy of Medical Sciences, Zhengzhou University, Zhengzhou 450052, China; 9 Department of Infectious Diseases, The First Affiliated Hospital of Zhengzhou University, Zhengzhou 450052, China; 10 State Key Laboratory of Environment Health (Incubation), Key Laboratory of Environment and Health, Ministry of Education, Key Laboratory of Environment and Health (Wuhan), Ministry of Environmental Protection, School of Public Health, Tongji Medical College, Huazhong University of Science and Technology, Wuhan 430030, China

**Keywords:** breast milk, N-acetylneuraminic acid, gut microbiota, bile acids, infant growth, germ-free mice

## Abstract

The healthy growth of infants during early life is associated with lifelong consequences. Breastfeeding has positive impacts on reducing obesity risk, which is likely due to the varied components of breast milk, such as N-acetylneuraminic acid (Neu5Ac). However, the effect of breast milk Neu5Ac on infant growth has not been well studied. In this study, targeted metabolomic and metagenomic analyses were performed to illustrate the association between breast milk Neu5Ac and infant growth. Results demonstrated that Neu5Ac was significantly abundant in breast milk from infants with low obesity risk in two independent Chinese cohorts. Neu5Ac from breast milk altered infant gut microbiota and bile acid metabolism, resulting in a distinct fecal bile acid profile in the high-Neu5Ac group, which was characterized by reduced levels of primary bile acids and elevated levels of secondary bile acids. Taurodeoxycholic acid 3-sulfate and taurochenodeoxycholic acid 3-sulfate were correlated with high breast milk Neu5Ac and low obesity risk in infants, and their associations with healthy growth were reproduced in mice colonized with infant-derived microbiota. *Parabacteroides* might be linked to bile acid metabolism and act as a mediator between Neu5Ac and infant growth. These results showed the gut microbiota-dependent crosstalk between breast milk Neu5Ac and infant growth.

## 1. Introduction

The healthy growth of infants during early life is associated with lifelong consequences. Being overweight or obese in childhood could increase the risk of adult adiposity and perhaps is associated with many health problems, such as type 1 diabetes and cardiovascular diseases [1,2]. It has been reported that infants with BMI z-scores greater than the 85th percentile of the World Health Organization standards were at risk of obesity [3,4]. Although genetics, early nutrition, lifestyle, and lack of physical activity are the direct factors leading to obesity, the potentially causal role of the early gut microbiota in childhood obesity has become increasingly prominent [5,6,7].

Sialylated oligosaccharides are some of the most important bioactive components in breast milk and could act as the prebiotics for the infant gut microbiota. In addition, sialylated oligosaccharides are associated with numerous benefits, such as promoting infant growth [8,9]. A previous study of two Malawian birth cohorts revealed that sialylated oligosaccharides could promote healthy infant growth in a microbiota-dependent manner in case of infant undernutrition [10]. Moreover, another study indicated that 3′-sialyllactose (3′-SL) plays a critical role in improving offspring’s health [11].

Sialic acid could be released from sialylated oligosaccharides by sialidases of gut microbiota [10,12]. Although most of the sialic acid is combined with oligosaccharides and proteins in breast milk, about 3% of the sialic acid still exists in free form and plays a very important role [13]. N-acetylneuraminic acid (Neu5Ac) is the predominant form of free sialic acid in humans, and 3′-SL and 6′-sialyllactose (6′-SL) are two abundant Neu5Ac-binding oligosaccharides in breast milk [9,14]. Moreover, the free Neu5Ac might reflect the metabolic status of total sialic acid or its availability because concentrations dropped with decreases in the oligosaccharide-bound and protein-bound forms of sialic acid in breast milk [13]. At present, there are many studies illustrating that free Neu5Ac is crucial for improving infant brain development and enhancing immunity [15]. However, the relationship between free Neu5Ac and the obesity risk of infants has not been clarified. Importantly, a high level of free Neu5Ac is one of the most discriminative characteristics of breast milk, when compared with baby formula [13]. Understanding the role of Neu5Ac in the growth of infants could be beneficial for developing prebiotics or supplements to improve growth outcomes.

In this study, we first focused on the relationship between the level of Neu5Ac in early breast milk and infant growth in later infancy in two independent Chinese infant cohorts. Next, by combining metagenomics and targeted metabolomic analyses, we illustrated the influences of breast milk Neu5Ac on the infant gut microbiota and the related metabolites and explored the interaction of the Neu5Ac-related metabolites with infant growth. Finally, the mediator role of the gut microbiota and bacterial-derived metabolites in the link between Neu5Ac and growth was validated in a gnotobiotic mouse model.

## 2. Materials and Methods

### 2.1. Study Cohorts and Sample Collection

Breast milk and neonatal fecal samples were collected from Chinese mother–newborn dyads during the first week after delivery between May and December 2018 in the cohort in a previous study (Zhengzhou cohort, *n* = 58) [16]. The fecal samples were collected within 24 h of breast milk sampling. The newborns (25 males/33 females) were all full-term and healthy. Seventeen newborns were delivered through C-section. All the samples were kept at −80 °C and analyzed within 1 year. Detailed responses to questionnaires of maternal and neonatal characteristics including general information, feeding pattern, and antibiotic usage are recorded in Table 1.

For the Wuhan cohort, only breast milk samples were collected around the first month of lactation and growth data were collected when infants were around 3 years old (*n* = 201). All the infants were full-term, and 131 of them were delivered by C-section. All the samples were kept at −80 °C and analyzed within 1 year. Detailed responses to questionnaires of maternal and neonatal characteristics and the infant growth indicators at around 3 years are shown in Table 1. The study was approved by the Ethics Committee of the First Affiliated Hospital of Zhengzhou University. Written informed consent was received from all mothers.

### 2.2. Assessment of Infant Growth

Infant growth indicators from the Zhengzhou cohort were collected at around 1 year of age, while infant growth data from the Wuhan cohort were collected at around 3 years of age. The infant BMI z-scores adjusted for age and sex were calculated under the guidance of World Health Organization standards in both of the cohorts [3]. Infants with BMI z-score greater than the 85th percentile were considered as the high obesity risk group [4], and the rest were grouped as the low obesity risk group. Specifically, growth data on 35 infants from the Zhengzhou cohort were collected, of whom 13 were classified as the high obesity risk group and 22 as the low obesity risk group. For the 201 infants from the Wuhan cohort, 118 infants were grouped as low obesity risk, and 83 infants were grouped as high obesity risk (Table 1).

### 2.3. Quantification of Neu5Ac and Sialylated Oligosaccharides in Breast Milk

Breast milk Neu5Ac, 3′-SL, and 6′-SL were quantified by an online solid-phase extraction–hydrophilic interaction chromatography (SPE-HILIC-MS) platform according to a previously used method [16]. The compound identification is based on the comparison with t_R_, *m*/*z*, and MS/MS fragments of the standards (Figure S1). In brief, 200 µL of breast milk samples was centrifuged at 8600× *g* for 20 min at 4 °C to eliminate lipids, and 200 µL of ethanol containing internal standards (Neu5Ac-^13^C6) was added to the skim breast milk to remove proteins. After centrifugation at 15,000× *g* for 15 min at 4 °C, 50 µL of supernatant was lyophilized, re-solubilized using 200 µL acetonitrile/water (*v*/*v* = 1/1), and quantified on the SPE-HILIC-MS platform. Finally, we obtained the Neu5Ac, 3′-SL, and 6′-SL concentrations of 58 breast milk samples from the Zhengzhou cohort and 201 samples from the Wuhan cohort.

### 2.4. Fecal Microbiota Transplantation to Germ-Free Mice

The experiment was conducted under the protocols approved by the Cyagen Biological Animal Ethics Committee. Specifically, approximately 500 mg of frozen feces from one infant with matched clinical information and infant age at sampling (10 days post-partum) from each group was suspended in 5 mL of reduced phosphate-buffered saline (PBS) in Hungate tubes. Germ-free C57BL/6 wild-type mice were colonized with the respective donor microbiota after a 4 h fasting by oral gavage of 200 µL of fecal slurry per mouse. The fecal slurry of the same infant was transplanted to 6 mice in parallel as a group. The rest of the fecal slurry was stored at −80 °C for the second gavage. Mice that received fecal microbiota from the same infant were housed together in the same cage (two mice per cage and six mice per group). The colonization was repeated the week after using the same fecal slurry. During the colonization, mice were fed sterile fodder and sterile water. Mice were housed in gnotobiotic facilities in 12 h day/night cycles during the whole experiment. Body weight was recorded every 3 days and mice were euthanized 2 weeks after the first oral gavage. The feces and cecal content were sampled and immediately put in liquid nitrogen. Gut microbial colonization in the gnotobiotic mice was assessed with about 20 mg of mice feces collected the day before the mice were euthanized by 16S rRNA gene sequencing analysis as previously described [16].

### 2.5. Metagenomic Analysis of the Newborn Gut Microbiota

Total DNA of newborn fecal samples was extracted and quantified with a previously described method [16]. Then, the DNA extract was fragmented to an average size of roughly 400 bp using Covaris M220 (Gene Company Limited, Hong Kong, China). Paired-end libraries were generated with NEXTFLEX Rapid DNA-Seq (Bioo Scientific, Austin, TX, USA) and sequenced by HiSeq Reagent Kits on an Illumina Hiseq (Illumina, San Diego, CA, USA) in accordance with manufacturer instructions.

Fastp (version 0.20.0) was used to trim adaptors and remove low-quality reads from the raw paired-end reads [17]. Next, BWA (version 0.7.9a) was used to align the reads to the human genome [18], and any hits associated with the reads and their mated reads were removed. MEGAHIT (version 1.1.2) was used to assemble the metagenomics data [19]. Contigs that were at least 300 bp were chosen as the final assembled result. MetaGene was applied to predict the open reading frames (ORFs) from each assembled contig [20]. A non-redundant gene catalog was built using CD-HIT (version 4.6.1) with 90% sequence identity and 90% coverage [21]. SOAPaligner (version 2.21) was used to map the reads to the non-redundant gene catalog with 95% identity after quality control [22], and gene abundance was evaluated and the relative abundance was normalized with TPM value as described previously [23].

The NCBI NR database was used for taxonomic annotation. Diamond (version 0.8.35) was used to align the representative sequences of the non-redundant gene catalog to the database with an e-value cutoff of 1 × 10^−5^ [24]. The genes that occurred in more than 5% of the samples were included in the following analysis [25]. The relative abundance of genes of the same genus was summed up to assess the relative abundance of the gut microbiota at the genus level. The Kyoto Encyclopedia of Genes and Genomes (KEGG) database was used for functional annotation by Diamond with an e-value cutoff of 1 × 10^−5^. The relative abundance of KEGG orthology (KO) was estimated by summing the relative abundance of genes of the same KO and renormalizing to one.

### 2.6. Bile Acid Analysis

For each sample, 100 mg of frozen infant fecal samples or 10 mg of frozen mice cecum content, a zirconia bead, and 1 mL of extraction solvent were mixed and homogenized with a mixed grinding apparatus (MM400, Retsch, Germany). The extraction solvent was composed of methanol with 0.3 μg/mL of cholic acid (CA)-d5, 0.9 μg/mL of chenodeoxycholic acid (CDCA)-d4, 0.6 μg/mL of glycocholic acid (GCA)-d5, 0.6 μg/mL of glycochenodeoxycholic acid (GCDCA)-d4, 0.3 μg/mL of taurocholic acid (TCA)-d5, and 0.3 μg/mL of taurodeoxycholic acid (TDCA)-d5. Next, the mixture was centrifuged at 4 °C, 14,000× *g* for 10 min to remove proteins. Then, 800 μL of the supernatant was lyophilized and reconstituted in 300 μL of acetonitrile/water (*v*/*v* = 1/1) for the following analysis.

An ultra-high-performance liquid chromatograph (UHPLC) coupled to a Shimadzu 8050 Triple Quad mass spectrometer (Shimadzu, Kyoto, Japan) with the electrospray ionization (ESI) source in negative ion mode was used for the targeted bile acid analysis. A Waters ACQUITY UPLC C8 column (1.7 µm, 2.1 × 100 mm) was used for separation. The column temperature was set as 40 °C and the flow rate was 0.2 mL/min. Mobile phase A was 10 mM NH_4_HCO_3_ aqueous solution and mobile phase B was acetonitrile. Multiple reaction monitoring (MRM) was used for mass analysis. The mass parameters were set as follows: nebulizing gas flow 3 L/min, heating gas flow 10 L/min, interface temperature 300 °C, heat block temperature 400 °C, and drying gas flow 10 L/min.

LabSolutions (version 5.89, Shimadzu, Kyoto, Japan) was used for instrument control and peak extraction. All the peak areas were corrected by internal standards and sample weight. Then, the absolute concentrations of bile acids were calculated by the external standard method and normalized by total sum scaling.

### 2.7. Statistical Analysis

All the box plots, bar plots, and scatter plots were visualized in GraphPad Prism 9.0 (GraphPad Software Inc., Boston, MA, USA). A Mann–Whitney U test was used for the significance test of continuous variables.

Beta diversity of the neonatal gut microbiota was calculated to evaluate the correlation between newborn gut genera and breast milk Neu5Ac content based on Bray–Curtis distance (RStudio 4.1.0, vegan package 2.5.7) and visualized in a principal coordinate analysis (PCoA) plot with the ggplot2 package (version 3.4.0), and adonis was conducted to evaluate the significant difference. Alpha diversity was calculated based on Simpson and Pielou indexes (RStudio 4.1.0, vegan package 2.5.7) to evaluate the gut microbial richness and evenness. Linear discriminant analysis effect size (LefSe) analyses were performed to assess the discriminative bacteria between different groups, and an LDR score above 2.0 was considered as significant [26]. Redundancy analysis (RDA) was conducted on the basis of the infant gut microbiota at the genus level on an online platform (http://cloud.biomicroclass.com/CloudPlatform) (accessed on 12 June 2023). Difference analysis of metabolic pathways was conducted with Welch’s *t*-test followed by FDR correction with the Benjamini–Hochberg method in STAMP [27], and a corrected *p*-value less than 0.05 was considered significant. Spearman correlation analysis was applied to estimate associations between genera and bacterial metabolic pathways in GraphPad Prism 9.0.

Levels of bile acids between different groups were compared by the logarithmic ratio of relative contents and a significant difference was defined as a combination of the fold change (FC) and *p*-value (|Log_2_ FC| > 0.6 and *p* < 0.05) and visualized in a volcano plot (ggpubr package 0.5.0, ggthemes package 4.2.4). Regression analysis was performed to evaluate the correlations between breast milk Neu5Ac level and infant growth and the correlation between the level of bile acids and infant growth or mouse growth in RStudio (lm function), and *p* < 0.05 was considered to show a significant difference. Random forest models were conducted as a feature selection technique to evaluate which bile acids were most important to differentiate samples based on infant growth (randomForest package, 4.7.1).

## 3. Results

### 3.1. Correlation between Breast Milk Neu5Ac Concentrations and Obesity Risk in Chinese Infants

**Figure 1:**
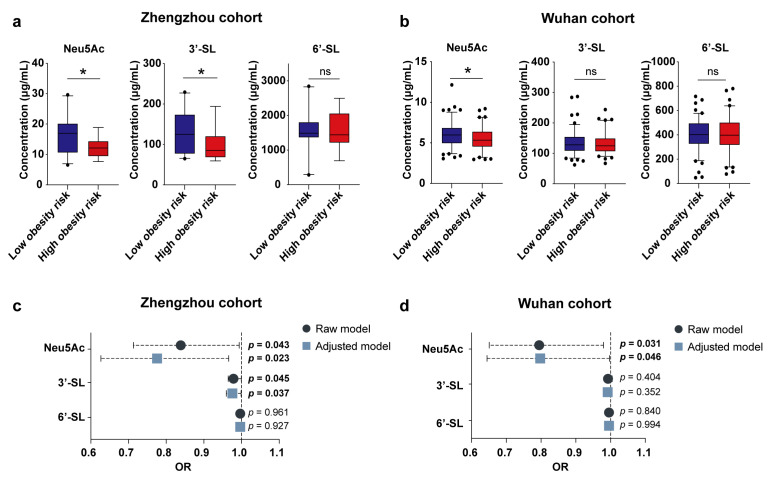
Correlation between breast milk Neu5Ac concentrations and the obesity risk of Chinese infants. (**a**,**b**) Concentrations of Neu5Ac, 3′-SL, and 6′-SL in breast milk of Chinese mothers collected from the Zhengzhou cohort (*n* = 35) (**a**) and Wuhan cohort (*n* = 201) (**b**), binned by the growth of their infants. Concentrations of Neu5Ac, 3′-SL, and 6′-SL are shown as mean ± SEM (* *p* < 0.05, ^ns^ *p* > 0.05, Mann–Whitney U test). (**c**,**d**) Regression analysis of breast milk Neu5Ac, 3′-SL, and 6′-SL concentrations and infant obesity risk in the Zhengzhou cohort (**c**) and Wuhan cohort (**d**). Adjusted confounders: infant sex, delivery mode, feeding pattern, and infant age at the time of breast milk sampling. OR, odds ratio; Neu5Ac, N-acetylneuraminic acid; 3′-SL, 3′-sialyllactose; 6′-SL, 6′-sialyllactose.

We first characterized the relationship between Neu5Ac/3′-SL/6′-SL in breast milk samples collected around 1 week post-partum and infant obesity risk at 1 year of age in the Zhengzhou cohort (*n* = 58). The results showed that high breast milk Neu5Ac and 3′-SL were both correlated with low obesity risk of the infants at the age of 1 year; that is, infants in the low obesity risk group were exposed to significantly higher levels of breast milk Neu5Ac and 3′-SL during the neonatal period (Figure 1a). Further adjusting for the confounders, including infant sex, delivery mode, feeding, and infant age at breast milk sampling, did not change the positive association between Neu5Ac/3′-SL and low obesity risk (Figure 1c).

Similar to the Zhengzhou cohort, early Neu5Ac content was significantly elevated in the breast milk of low obesity risk infants aged 3 years old in the Wuhan cohort (Figure 1b). Correction for confounding factors did not alter the correlation between Neu5Ac and infants’ obesity risk (Figure 1d). Meanwhile, the association between 3′-SL and infant obesity risk was not observed in the Wuhan cohort. Altogether, these results from the two independent Chinese cohorts suggested that early breast milk Neu5Ac is important for reducing the obesity risk of infants later in infancy.

### 3.2. Gut Microbiota-Dependent Association between Breast Milk Neu5Ac and Growth

Neu5Ac is involved in the establishment of newborn gut microbiota, which is closely related to the growth of infants later. To further explore the role of gut microbiota in the correlation between Neu5Ac and infant growth, fecal samples of the infants from the Zhengzhou cohort were collected within 24 h of breast milk sampling (*n* = 58) and a fecal microbiota transplantation (FMT) experiment was conducted in germ-free mice to verify the mediating role of microbiota between Neu5Ac concentration and growth.

**Figure 2:**
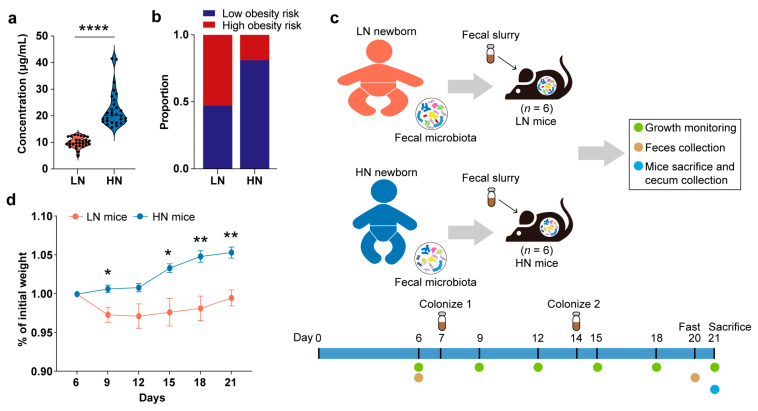
Effect of gut microbiota in infants fed with breast milk with different Neu5Ac levels on the growth of gnotobiotic mice. (**a**) The concentration of breast milk Neu5Ac in the LN group and HN group. Concentration is shown as mean ± SEM (**** *p* < 0.0001, Mann–Whitney U test). (**b**) Ratio of low obesity risk infants to high obesity risk infants between LN group and HN group. (**c**) Design of gnotobiotic mouse experiments. (**d**) Weight gain normalized to body weight of LN mice and HN mice (** *p* < 0.01, * *p* < 0.05, Mann–Whitney U test). LN mice: mice colonized with the feces from the infants in the LN group; HN mice: mice colonized with the feces from the infants in the HN group.

First, the infants in the Zhengzhou cohort were divided into the low-Neu5Ac (LN) group (*n* = 29) and high-Neu5Ac (HN) group (*n* = 29) according to the median concentration of Neu5Ac in their breast milk (Table 2). The LN group included those infants with breast milk Neu5Ac concentrations below the median, and the HN group included those infants with breast milk Neu5Ac concentrations above the median. The HN group was exposed to breast milk Neu5Ac concentrations 2.5 times higher than that of the LN group (*p* < 0.0001, Mann–Whitney U test) (Figure 2a). Moreover, the proportion of low obesity risk infants was higher in the HN group (Figure 2b).

Next, a fecal sample from one infant with matched clinical information from each of the LN and HN groups was taken separately to make a fecal slurry (Table S1), then transplanted into young (5-week-old) male germ-free C57BL/6 mice by oral gavage. The fecal slurry of the same infant was transplanted into 6 mice in parallel as a group (Figure 2c). After the oral gavage of fecal slurry was administered twice, and two weeks of growth monitoring was carried out, we found that mice colonized with fecal microbiota from the HN infants had stable weight gain, while mice colonized with the fecal bacteria from the LN group showed a dramatic weight loss followed by a slow weight gain until the mice were sacrificed (Figure 2d). Additionally, two of the LN fecal bacteria-colonized mice exhibited ruffled back fur after the first colonization, suggesting a sub-healthy state of the mice. Collectively, we speculated that breast milk Neu5Ac-related gut microbiota is involved in the regulation of healthy growth.

### 3.3. Effect of Breast Milk Neu5Ac on the Composition and Function of Gut Microbiota

To further explore the specific role of breast milk Neu5Ac in the colonization of infant gut microbiota from early life, metagenomic sequencing analysis was performed on the fecal samples collected in parallel with the breast milk to profile the gut microbiota of infants from the Zhengzhou cohort, and the effects of breast milk Neu5Ac on gut microbiota were characterized. In total, we generated 243.28 GB of paired-end reads of high-quality sequences (average 4.19 Gb per sample).

**Figure 3:**
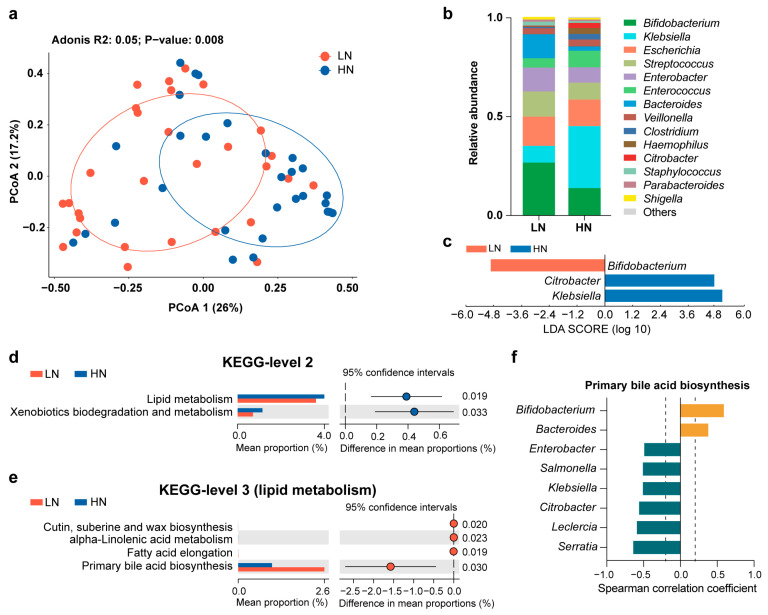
Effects of breast milk Neu5Ac on the composition and function of the infant gut microbiota in the Zhengzhou cohort. (**a**) Principal coordinate analysis (PCoA1 and PCoA2) of the infant gut microbiota at the genus level based on Bray–Curtis distance, with community structure differences tested by adonis analysis of variance with 999 permutations. (**b**) Comparisons of the mean relative abundance of gut microbiota at the genus level between the LN group and HN group. Genera with relative abundance above 1% are shown in the bar plot. “Others” indicates sum of the bacteria with relative abundance less than 1%. (**c**) Discriminative bacteria between LN group and HN group. Genera with relative abundance above 1% are included in the analysis. (**d**) Differences in the microbial metabolic pathway based on level 2 of the KEGG database. (**e**) Differences in microbial lipid metabolic pathway of the KEGG database (level 3). (**f**) Spearman correlation coefficients (r) between gut genera and primary bile acid biosynthesis pathway. Orange indicates a positive correlation and green indicates a negative correlation. Genera with relative abundance above 1% are included in the analysis. Significant correlations with |r| > 0.2 and *p* < 0.05 are shown.

**Figure 4:**
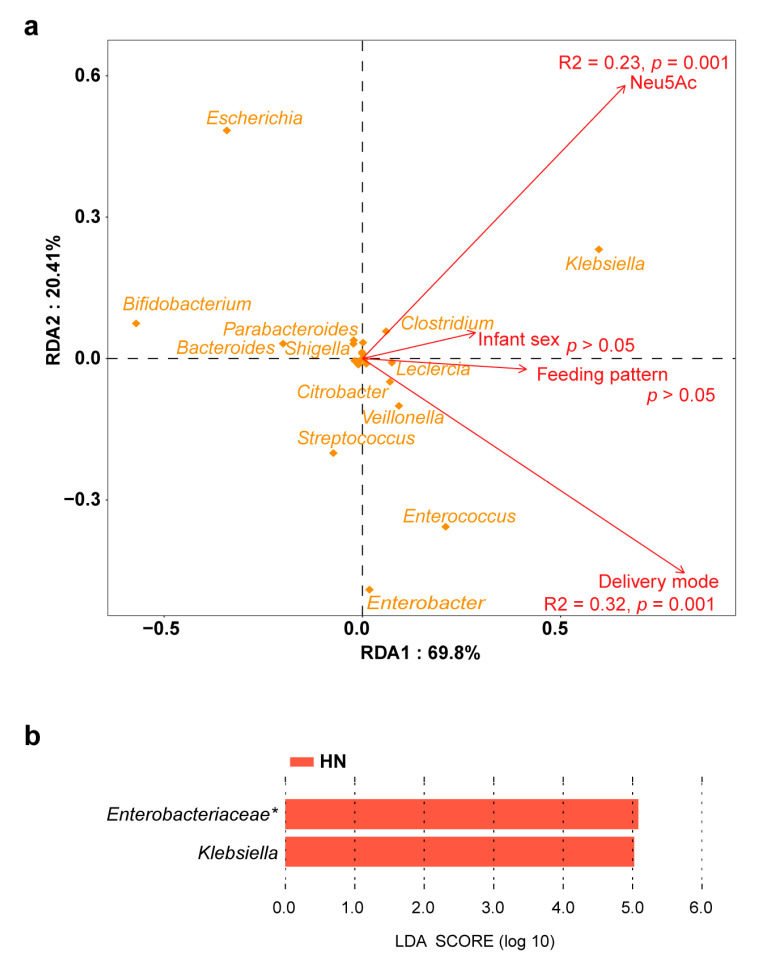
Influencing factors of gut microbiota in the Zhengzhou cohort. (**a**) Redundancy analysis of impact factors on the infant gut microbiota. (**b**) Discriminative bacteria between LN group and HN group in the vaginally delivered infants. Genera with relative abundance above 1% are included in the analysis. Asterisks (*) indicates the unclassified bacteria at the genus level.

Clear separation of the gut microbiota was observed between the LN group and HN group (Bray–Curtis distance, adonis, permutations = 999, *p* = 0.008) (Figure 3a). However, breast milk Neu5Ac levels did not cause a change in alpha diversity between the two groups (Figure S2). In terms of the gut microbiota composition, only a few bacteria were associated with Neu5Ac content. For instance, *Bifidobacterium* was abundant in the LN group and *Klebsiella* was enriched in the HN group (Figure 3b,c). Similarly, this difference in microbial composition was also present in germ-free mice colonized with fecal microbiota from the LN and HN groups, although the gut microbial richness and evenness were higher in the LN group (Figure S3a–d). Notably, breast milk Neu5Ac was not the unique impact factor on the infant gut microbiota, delivery mode also had an impact on the gut microbial community according to the RDA (Figure 4a). Therefore, the effect of breast milk Neu5Ac levels on the composition of gut microbiota in vaginally delivered infants of the Zhengzhou cohort was further analyzed, and the enrichment of *Klebsiella* in the HN group could also be observed (Figure 4b).

Further analysis based on the functional capacity of the infant gut microbiota indicated that lipid metabolism, especially bile acid metabolism, was highly correlated with breast milk Neu5Ac (Figure 3d,e). Moreover, the discriminative bacteria between HN and LN groups exhibited different levels of involvement in primary bile acid biosynthesis (Figure 3f). For instance, *Bifidobacterium* and *Bacteroides*, the LN group’s abundant bacteria, were positively associated with primary bile acid biosynthesis, while *Klebsiella*, the bacterium enriched in the HN group, was negatively correlated with primary bile acid biosynthesis. Altogether, these findings suggested the presence of an interaction between Neu5Ac and gut microbial bile acid metabolism.

### 3.4. Conjugated Bile Acids in the Correlations between Breast Milk Neu5Ac and Infant Obesity Risk

**Figure 5:**
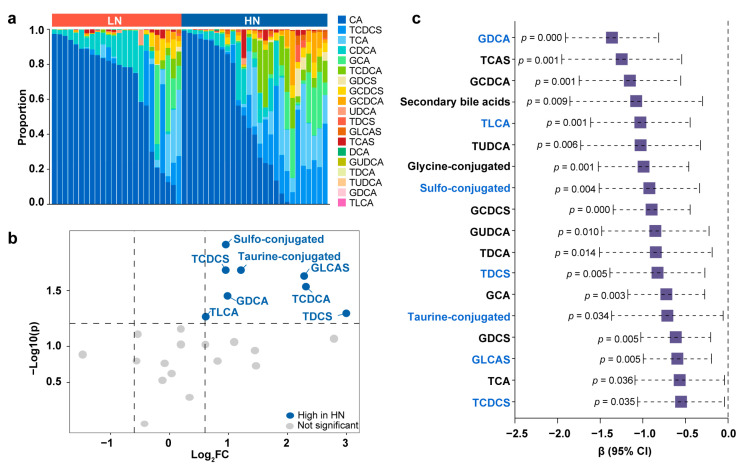
Linkage of conjugated bile acids in the correlations between breast milk Neu5Ac and infant growth. (**a**) Fecal bile acid composition in newborns of LN group and HN group (GDCS, glycodeoxycholic acid 3-sulfate; GCDCS, glycochenodeoxycholic acid 3-sulfate; UDCA, ursodeoxycholic acid; TCAS, taurocholic acid 3-sulfate; GUDCA, glycoursodeoxycholic acid; TUDCA, tauroursodeoxycholic acid). (**b**) Volcano plot of different bile acids between LN group and HN group. Fold changes were calculated as the ratio of each bile acid between the HN group and LN group and converted logarithmically. Blue indicates Log_2_ FC > 0.6 and *p* < 0.05 (Mann–Whitney U test), and grey indicates no significant difference. See also Table S2. (**c**) Regression analysis to estimate the associations between the newborn fecal bile acids and infant BMI aged 12 months. Linear regression models were controlled for the following confounders: infant sex, delivery mode, feeding pattern, and infant age at sampling. Bile acids marked with blue are those breast milk Neu5Ac-related bile acids identified in (**b**).

Next, targeted tandem mass spectrometry was used to measure the levels of 19 bile acids of fecal samples in the Zhengzhou cohort, and the distinct bile acid profile was characterized between the LN group and HN group. In general, a higher proportion of primary unconjugated bile acids, namely, CA and CDCA, was found in the LN group (Figure 5a). Primary unconjugated bile acids can be converted into secondary or conjugated bile acids by gut microbiota. We observed that the HN group contained a greater abundance of conjugated bile acids, especially taurine- and sulfo-conjugated deoxycholic acid (DCA), CDCA, and lithocholic acid (LCA) (Figure 5b and Table S2).

Notably, many of the discriminative bile acids between the LN group and HN group were significantly correlated with infant obesity risk later in life. In regression models adjusting for the confounders (infant sex, delivery mode, feeding pattern, and infant age at breast milk sampling), the total proportions of sulfo-, taurine-, and glycine-conjugated bile acids all inversely contributed to a high BMI in infants at 12 months of age (Figure 5c). Looking at the individual bile acids, glycodeoxycholic acid (GDCA), taurolithocholic acid (TLCA), glycolithocholic acid (GLCA), and taurochenodeoxycholic acid 3-sulfate (TCDCS) were the main bile acids that were related to infant growth. Consistently, the random forest models using the newborn bile acid content to predict infant obesity risk had an out-of-bag error rate of 13.79%, and the most important bile acids contributing to the model were TLCA, GDCA, and glycolithocholic acid 3-sulfate (GLCAS) (Figure S4).

To further explore the association of infant obesity risk with the bile acid levels in newborns, the above Neu5Ac-related bile acids were classified in quartiles and logistic regression analyses were performed. A higher relative abundance of TLCA was associated with a 91.5% decrease in high obesity risk (above vs. below median, OR = 0.085, 95% CI, 0.013–0.542, *p* = 0.007). An increased level of GDCA was correlated with a 93.7% decrease in high obesity risk (above vs. below median, OR = 0.063, 95% CI, 0.010–0.421, *p* = 0.004). Elevation of TCDCS was associated with an 85.9% decrease in high obesity risk (above vs. below median, OR = 0.141, 95% CI, 0.023–0.857, *p* = 0.033). Additionally, the enrichment of taurodeoxycholic acid 3-sulfate (TDCS) or GLCAS tended to be correlated with high obesity risk (above vs. below median, OR = 0.239, 95% CI, 0.047–1.219, *p* = 0.085). Taken together, breast milk Neu5Ac may be correlated with gut microbial bile acid metabolism, which in turn is associated with infant obesity risk.

### 3.5. Validation of the Interactions among Neu5Ac, Gut Microbiota, Bile Acid Metabolism, and Healthy Growth in Gnotobiotic Mice

**Figure 6:**
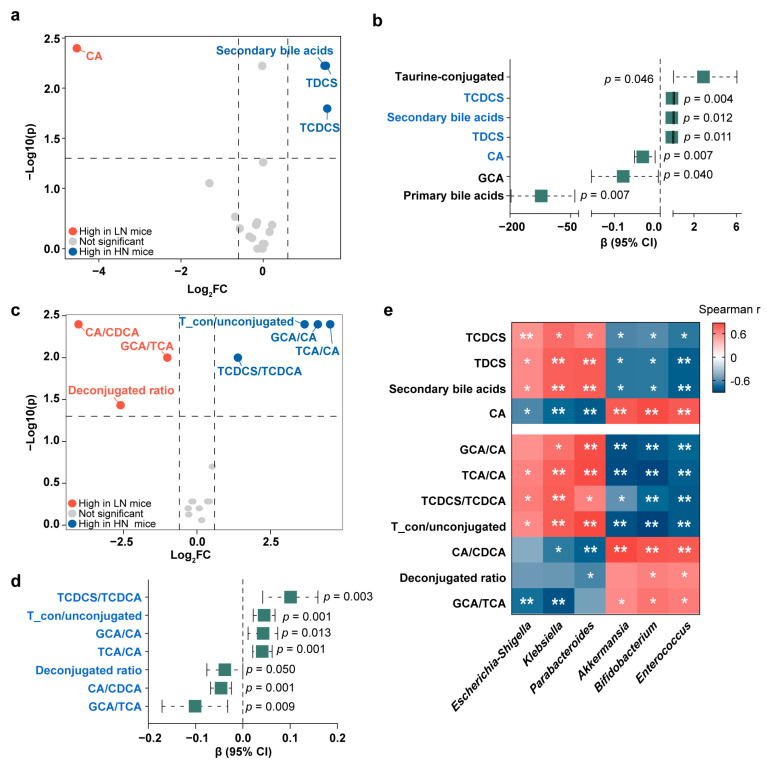
Validation of the association between bile acid and healthy weight gain in mice. (**a**) Volcano plot of different bile acids between LN mice and HN mice. (**b**) Regression analysis to estimate the associations between the mouse cecal bile acid content and weight gain. (**c**) Volcano plot of bile acid conversion to evaluate the enzymatic activity of bile acid metabolism between LN mice and HN mice. (**d**) Regression analysis to estimate the associations between the mouse cecal bile acid conversion and weight gain. (**e**) Heatmap of Spearman correlation coefficients between mouse cecal bile acids and gut microbiota at the genus level (* *p* < 0.05 and ** *p* < 0.01). Fold changes were calculated as the ratio of each bile acid between HN mice and LN mice and converted logarithmically. Blue indicates Log_2_ FC > 0.6 and *p* < 0.05 and red indicates Log_2_ FC < −0.6 and *p* < 0.05, and grey indicates no significant difference (Mann–Whitney U test). See also Tables S3 and S4. Bile acids marked with blue are discriminative bile acids between LN mice and HN mice identified in (**a**) or (**c**).

We next examined whether the above observed correlations of breast milk Neu5Ac with bile acid metabolism and healthy growth in infants also occurred in the FMT mice. Similar to the Zhengzhou cohort, two sulfo-conjugated bile acids, TDCS and TCDCS, were enriched in the mice colonized with the fecal bacteria from the HN group (Figure 6a and Table S3), suggesting that the differences in bile acids were related to gut microbiota. Just as mentioned above, mice colonized with the bacteria from the HN infants exhibited a healthier growth, and our results showed that the HN group’s abundant bile acids, TDCS and TCDCS, may have contributed to this (Figure 6b).

Additionally, by calculating the ratios of bile acids, we analyzed the activities of enzymes involved in bile acid metabolism and found a significant elevation of GCA/CA, TCA/CA, and TCDCS/taurochenodeoxycholic acid (TCDCA) in the HN mice (Figure 6c and Table S4), suggesting that enzymes involved in the conjugation of bile acids were elevated in the HN mice. Notably, GCA/CA, TCA/CA, and TCDCS/TCDCA were all found to be associated with favorable growth in the mice (Figure 6d).

Finally, we found that the HN group-related bacteria, *Klebsiella* and *Parabacteroides*, were positively associated with the healthy growth-correlated bile acids (Figure 6e). Specifically, *Klebsiella* and *Parabacteroides* were both positively correlated with TDCS and TCDCS and negatively correlated with CA. The positive associations of *Klebsiella* and *Parabacteroides* with conjugated bile acids further indicated that the enzymes that facilitates bile acid conjugation may be highly expressed in these bacteria.

## 4. Discussion

Based on the two independent cohorts comprising different lactation periods of breast milk samples from Chinese mothers, our study indicated that the milk from mothers of children exhibiting low obesity risk in later infancy contained a higher level of Neu5Ac in early lactation. Approximately 73% of the Neu5Ac in breast milk is conjugated with oligosaccharides to generate sialylated oligosaccharides, while there is still small but significant amount of Neu5Ac existing in free form, much higher than in formula milk [13]. Previous studies have proposed that breast milk sialylated oligosaccharides were related to infant growth in both full-term and pre-term infants [10,28,29]. Consistently, our study showed that the Neu5Ac in breast milk was also associated with reduced obesity risk in infants. The concentration of Neu5Ac in breast milk changed across lactation [29], and our study further indicated that Neu5Ac levels in breast milk collected one week post-partum (colostrum and transitional milk) and one month post-partum (mature milk) might both affect growth later in infancy. Infant sex, delivery mode, feeding, and infant age at breast milk sampling might be the impact factors of both breast milk Neu5Ac/3′-SL concentration and infant growth [9,30,31,32], and we showed that adjusting for the above confounders did not change the positive association between Neu5Ac/3′-SL and low obesity risk. The Wuhan cohort and the Zhengzhou cohort are two independent cohorts composed of breast milk samples from different periods, and the similar associations between Neu5Ac and infant obesity risk from the above two cohorts raised the possibility that Neu5Ac plays an important role in infant growth. Although the liver has the ability to de novo synthesize sialic acid from glucose, the activity of the rate-limiting enzyme (UDP-N-acetylglucosamine-2-epimerase) is low during the neonatal period [33], indicating that breast milk Neu5Ac is an important source for newborns, and dietary supplementation with Neu5Ac might be beneficial for infants who cannot be breastfed.

Neu5Ac is a nine-carbon monosaccharide that plays a role in shaping infant gut microbiota. *Bifidobacterium* expresses sialidase that could liberate sialic acids from sialyloligosaccharides, gangliosides, and glycoproteins [34]. The enrichment of *Bifidobacterium* in the LN group might make up the deficiency of sialic acid content in breast milk. *Klebsiella* could utilize sialic acid as a carbon source as, consistently, high breast milk Neu5Ac was related to abundant *Klebsiella* [35]. A previous study indicated that sialyllactose, a conjugated form of Neu5Ac, tended to interplay with the gut bacterial transcriptional function more than composition [10]. As a microbial metabolite of sialyllactose [10], we noted that Neu5Ac was also intensely correlated with the metabolic function of gut microbiota. Gut bacterial lipid metabolism, especially bile acid metabolism, varied considerably with the level of breast milk Neu5Ac. Bile acids are some of the most important kinds of gut microbial metabolites. They are synthesized in the liver from cholesterol through two different metabolic pathways, namely, the classic pathway and the alternative pathway. Primary bile acids comprising CA and CDCA are products of the above two pathways, respectively [36]. CA and CDCA are conjugated to either taurine or glycine in the liver, secreted into the bile, and released into the duodenum after ingestion of food. Once in the gut, bile acids are transformed by gut microbiota to produce a wide variety of secondary bile acids [37]. The wide array of secondary bile acids in the HN group suggested that the gut microbiota of that group possessed more abundant bile acid metabolic function. Sulfonation of bile acids increases their solubility and enhances their fecal excretion, and elevated levels of sulfo-conjugated bile acids have been detected in fecal samples of breastfed infants [38,39]. The content of Neu5Ac in breast milk is much higher than that in formula milk [13], and the positive associations between breast milk Neu5Ac and sulfo-conjugated bile acids identified in our study suggested that higher Neu5Ac content in breast milk might contribute to the elevated levels of sulfo-conjugated bile acids in breastfed infants. Collectively, reduced levels of primary bile acids and elevated levels of secondary bile acids have been reported as a feature of healthy individuals [40,41], which was consistent with the characteristics of the fecal bile acid pool in the HN group infants, who displayed lower obesity risk.

Bile acids are key regulators in maintaining the energy balance and metabolic homeostasis of the host [42,43]. It has been reported that dysregulated bile acid metabolism is associated with growth faltering or obesity [44,45], and our study further proposed that the microbiota-derived bile acids might be the mediators in the complicated interaction between breast milk Neu5Ac and infant growth. In our study, several bile acids belonging to conjugated CDCA, DCA, and LCA were elevated in the HN group and were potentially related to reducing the obesity risk of the infants from that group. Consistently, a previous study has reported that levels of CDCA and DCA were correlated with activities of energy metabolism enzymes, such as gastrointestinal hormones, pancreatic peptide YY, and glucagon-like peptide-1 (GLP-1) [46]. Moreover, CDCA, DCA, and LCA have been identified as signaling molecules for the activation of farnesoid X receptor (FXR) [47]. By binding to the intestinal FXR, bile acids can induce the expression of endocrine hormone fibroblast growth factor 19 (FGF19), which suppresses lipogenesis and increases fatty acid oxidation in the liver, thereby regulating body weight gain and reducing the risk of obesity [48].

Although humans and mice metabolize Neu5Ac in different ways, and its main derivatives are also different [12,49], the aim of the study was to explore the association between Neu5Ac-related gut microbiota and infant growth, thus germ-free mice were used for colonization of infant gut microbiota whose breast milk has different levels of Neu5Ac. Germ-free mice are leaner than conventionally raised mice [50]. The healthy germ-free mice would gain weight after colonization with gut microbiota from infants [51]. Thus, the stable weight gain of HN mice indicated that mice transplanted with fecal bacteria from the HN infants exhibited a healthier growth, which is in line with the fact that the HN infants presented healthier growth due to a low risk of obesity. Therefore, the similar healthy growth of HN infants and HN mice provided additional evidence that the crosstalk between breast milk Neu5Ac and infant growth is gut microbiota-dependent. Further bile acid analysis of the gnotobiotic mice validated the idea that gut microbial bile acid metabolism is important in the weight regulation of the mice after FMT. As bile acid composition in mice is somewhat distinct from that in humans [52], a total of 14 bile acids identified in the infants were focused on in the FMT mice. A previous report showed that the cecum bile acid pool in mice was mainly composed of primary bile acids [53], and the majority of the bile acids identified in the mouse models of our study were taurine-primary bile acids. Conventionalization of germ-free C57BL/6 mice with a normal microbiota produces an increase in both body weight and body fat [51,54]. Our study consistently suggested that two taurine-conjugated bile acids in HN infants, TDCS and TCDCS, were linked with healthy weight gain in the mice after FMT. Bile acid regulation pathways in humans and mice are remarkably similar, that is, CDCA and DCA could engage FXR and activate expression of FGF15, thus having a myriad of other effects including regulation of lipogenesis and metabolic rate in mice [55]. Next, we found that two discriminative bacteria in the HN group, *Klebsiella* and *Parabacteroides*, were correlated with the level of the growth-regulating bile acids. It has been well established that *Parabacteroides* is capable of producing secondary bile acids [56] and thus may be able to alleviate obesity and metabolic dysfunctions [57]. However, no reports on the metabolism of bile acids by *Klebsiella* have been published so far. Therefore, we speculated that the correlation of *Klebsiella* with bile acids reflects that certain bacteria interacting with *Klebsiella* might be the primary metabolizers of bile acids, while *Klebsiella* benefits secondarily. Clearly, further investigation into the roles of *Klebsiella* and its related bacteria in bile acid metabolism is warranted.

However, this study still has several limitations. First, some of the infants included in this study were delivered by cesarean section or were subjected to mixed feeding, both of which are important factors affecting infant growth. Although adjusting for these factors in the regression model did not affect the correlation between breast milk Neu5Ac and infant growth in the two cohorts, future studies should validate the results of this study using vaginally delivered and breastfed infants. Second, our study focused only on Chinese cohorts, so it is unclear whether our findings can be generalized to infants in different countries. Next, despite our analysis of breast milk specimens from Chinese mothers revealing an association between Neu5Ac abundance and infant growth, additional time-series studies of mother–infant dyads from other cohorts are needed to determine the extent to which breast milk Neu5Ac-related variations in infant microbiota composition and function correlate with infant growth outcomes.

## 5. Conclusions

Our study demonstrated that breast milk Neu5Ac was associated with infant obesity risk in a gut microbiota-dependent manner. Breast milk Neu5Ac altered gut microbiota and reprogrammed bile acid metabolism, resulting in a distinct fecal bile acid profile in the HN group, which was characterized by reduced levels of primary bile acids and elevated levels of secondary bile acids. The conjugated DCA and CDCA were elevated in the HN group and positively correlated with reducing infant obesity risk. Especially, two sulfo- and taurine-conjugated bile acids, TDCS and TCDCS, were Neu5Ac-related and they were also helpful for healthy growth promotion, and the associations with healthy growth were reproduced in mice colonized with infant-derived microbiota. Finally, we proposed that *Parabacteroides* might be involved in bile acid metabolism and act as the mediator between Neu5Ac and infant growth. Additional studies are needed to clarify the mechanism of specific gut bacterial species and the correlated bile acids that contribute to early life growth and development. Our study might help to identify strategies to develop prebiotics to improve the growth outcomes of infants.

## Figures and Tables

**Table 1:** Characteristics of the mothers and infants included in the study.

	Zhengzhou Cohort (*n* = 58)	Wuhan Cohort (*n* = 201)
Sampling age of infant (days)		
Breast milk	4 (3–7)	41 (33–43)
Newborn feces	5 (3–7)	Not collected
Maternal characteristics		
Gestational age (day)	277 (272–281)	273 (266–280)
Delivery mode		
Natural delivery	41	70
C-section	17	131
Maternal antibiotic usage ^1^	23	No information
Maternal BMI (kg/m^2^) ^2^		
Pre-pregnancy	20.26 (18.81–22.63)	20.06 (18.99–21.64)
Pre-delivery	27.07 (24.57–29.55)	27.32 (24.80–29.24)
Neonatal characteristics		
Infant sex ^3^		
Male	25	111
Female	33	83
Feeding pattern ^4^		
Mostly breastmilk feeding	18	102
Mixed feeding	40	98
Growth indicator ^5^		
Infant age at time of BMI recording (months)	12 (12–13)	30 (24–36)
Infant BMI	16.98 (16.01–18.41)	16.78 (15.52–17.72)
Low obesity risk (No.)	22	118
High obesity risk (No.)	13	83

^1^ There were 4 missing data in the Zhengzhou cohort; ^2^ 14 missing data in the Zhengzhou cohort and 18 missing data in the Wuhan cohort; ^3^ 4 missing data in the Wuhan cohort; ^4^ feeding pattern at the time of breast milk sampling. There were 1 missing datum in the Wuhan cohort; ^5^ 23 missing data in the Zhengzhou cohort. Growth indicator data from the Zhengzhou cohort and Wuhan cohort were collected when the infants were 1 year old and 3 years old, separately. All the continuous variables are shown in median and interquartile ranges.

**Table 2:** Clinical characteristics of the infants in the LN group and HN group in the Zhengzhou cohort.

	LN Group (*n* = 29)	HN Group (*n* = 29)
Sampling age of infant (days)		
Breast milk	6 (4–13)	4 (3–5)
Newborn feces	5 (4–13)	4 (3–5)
Maternal characteristics		
Gestational age (day)	277 (274–280)	273 (270–284)
Delivery mode		
Natural delivery	25	16
C-section	4	13
Maternal antibiotic usage ^1^	8	15
Maternal BMI (kg/m^2^) ^2^		
Pre-pregnancy	19.53 (18.78–22.03)	21.57 (19.53–23.42)
Pre-delivery	26.30 (24.45–28.40)	27.55 (26.02–30.20)
Neonatal characteristics		
Infant sex		
Male	13	12
Female	16	17
Feeding pattern ^3^		
Mostly breastmilk feeding	10	8
Mixed feeding	19	21
Growth indicator ^4^		
Infant age at time of BMI recording (months)	13 (12–13)	12 (12–13)
Infant BMI	17.97 (16.01–18.66)	16.64 (16.00–17.12)
Low obesity risk (No.)	9	13
High obesity risk (No.)	10	3

^1^ There were 1 missing datum in LN group and 3 missing data in HN group; ^2^ 4 missing data in LN group and 10 missing data in HN group; ^3^ feeding pattern at the time of breast milk sampling; ^4^ 10 missing data in LN group and 13 missing data in HN group. Growth indicator data from the Zhengzhou cohort were collected when the infants were 1 year old. All the continuous variables are shown in median and interquartile ranges.

## Data Availability

The data for this study have been deposited in the European Nucleotide Archive (ENA) at EMBL-EBI under accession number PRJEB 57911.

## Supplementary Materials

The following supporting information can be downloaded at: https://www.mdpi.com/article/10.3390/metabo13070846/s1, Figure S1: The MS/MS spectra of Neu5Ac in standard sample and breast milk sample; Figure S2: Box plots of alpha diversity evaluated by Simpson and Pielou indexes between LN and HN group; Figure S3: Gut microbial composition of the gnotobiotic mice 2 weeks after fecal microbiota transplantation; Figure S4: Important bile acids from random forests for classifying low obesity risk and high obesity risk infants in the Zhengzhou cohort; Table S1: Clinical information of the donors for fecal microbiota transplantation; Table S2: Fold change of fecal bile acids between LN and HN infants; Table S3: Fold change of cecal bile acids between LN and HN mice; Table S4: Fold change of cecal bile acid conversion between LN and HN mice.
