# Supplementary material for: Crosstalk between Breast Milk N-Acetylneuraminic Acid and Infant Growth in a Gut Microbiota-Dependent Manner
Source: Metabolites. 2023 Jul 13;13(7):846. doi: 10.3390/metabo13070846 (PMC10385641; doi:10.3390/metabo13070846)
Supplement: Supplementary file 1 [file metabolites-13-00846-s001.zip › metabolites-2477292-supplementary.pdf]

Supplementary Materials

Crosstalk between breast milk N-acetylneuraminic acid and infant growth in a gut microbiota-dependent manner

Runze Ouyang [1,2,3], Sijia Zheng [1,2,3], Xiaolin Wang [1,3], Qi Li [1,3], Juan Ding [4] , Xiao Ma [5], Zhihong Zhuo [6], Zhen Li [7], Qi Xin [8], Xin Lu [1,2,3], Lina Zhou [1,2,3], Zhigang Ren [9], Surong Mei [10], Xinyu Liu [1,2,3,]* and Guowang Xu [1,2,3]

[1] CAS Key Laboratory of Separation Science for Analytical Chemistry, Dalian Institute of Chemical Physics, Chinese Academy of Sciences, Dalian 116023, Liaoning, China; ouyangrunze@dicp.ac.cn (R.O.); zhengsj@dicp.ac.cn (S.Z.); xugw@dicp.ac.cn (G.X.)

[2] University of Chinese Academy of Sciences, Beijing 100049, China; luxin001@dicp.ac.cn (X.L. (Xin Lu)); zhouln@dicp.ac.cn (L.Z.)

[3] Liaoning Province Key Laboratory of Metabolomics, Dalian 116023, Liaoning, China; wangxiaolin@dicp.ac.cn (X.W.); liqi1808@dicp.ac.cn (Q.L.)

[4] Department of Quality Control, The First Affiliated Hospital of Zhengzhou University, Zhengzhou 450052, Henan, China; dingjuan241@163.com

[5] Department of Nursing, The First Affiliated Hospital of Zhengzhou University, Zhengzhou 450052, Henan, China; maxiao199304@163.com

[6] Department of Pediatric, The First Affiliated Hospital of Zhengzhou University, Zhengzhou 450052, Henan, China; zhuozhihong@126.com

[7] Department of Interventional Radiology, The First Affiliated Hospital of Zhengzhou University, Zhengzhou 450052, Henan, China; lizhenjrfs@zzu.edu.cn

[8] Academy of Medical Sciences, Zhengzhou University, Zhengzhou 450052, Henan, China; 202011401030361@gs.zzu.edu.cn

[9] Department of Infectious Diseases, The First Affiliated Hospital of Zhengzhou University, Zhengzhou 450052, Henan, China; fccrenzg@zzu.edu.cn

[10] State Key Laboratory of Environment Health (Incubation), Key Laboratory of Environment and Health, Ministry of Education, Key Laboratory of Environment and Health (Wuhan), Ministry of Environmental Protection, School of Public Health, Tongji Medical College, Huazhong University of Science and Technology, Wuhan 430030, Hubei, China; surongmei@hust.edu.cn

* Correspondence: liuxy2012@dicp.ac.cn

Supplementary Figures

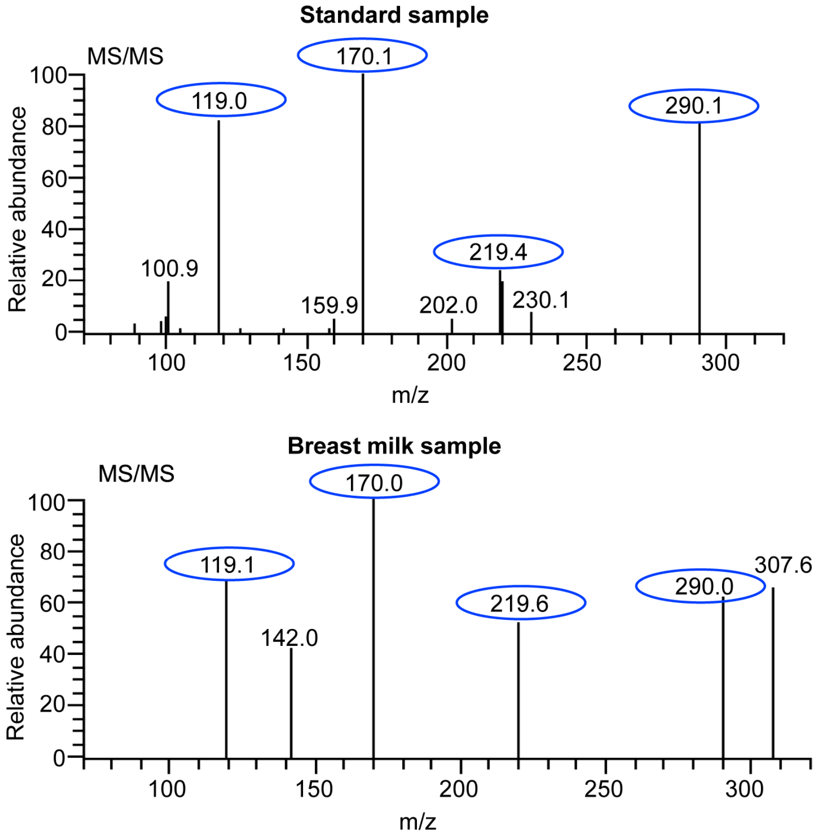

Figure S1. The MS/MS spectra of Neu5Ac in standard sample and breast milk sample. Main fragments matched well and were circled in oval.

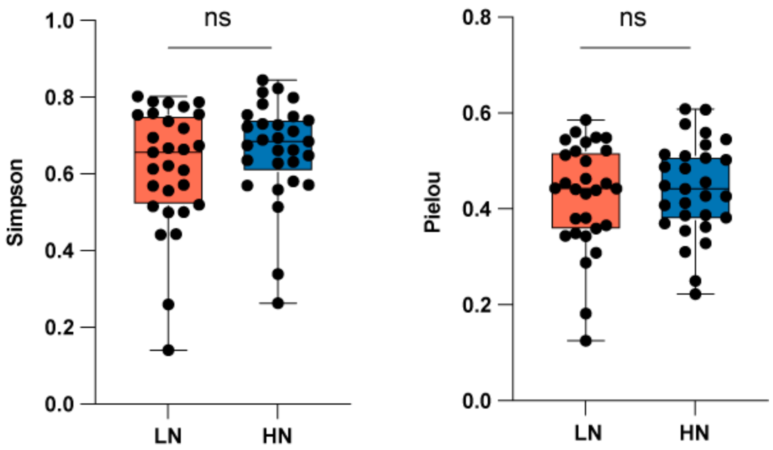

Figure S2. Boxplots of alpha diversity evaluated by Simpson and Pielou indexes between LN and HN group ($^{ns}p > 0.05$, Mann-Whitney U test). LN: Low Neu5Ac group, HN: High Neu5Ac group.

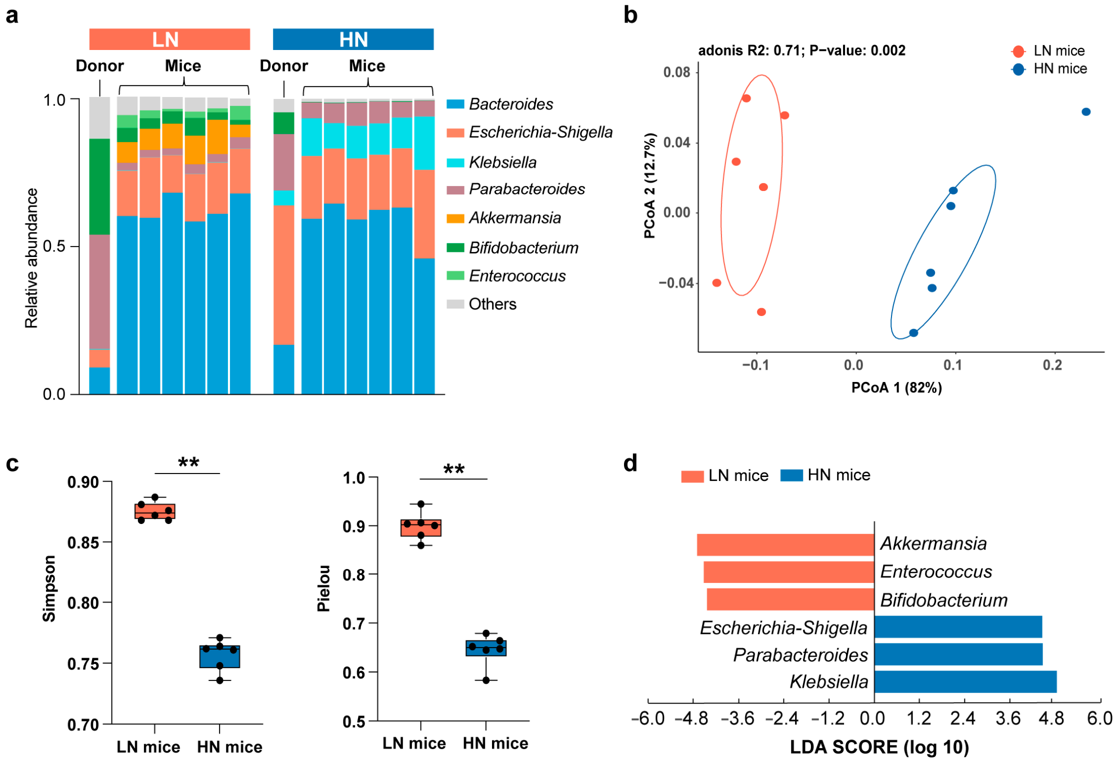

Figure S3. Gut microbial composition of the gnotobiotic mice after 2 weeks of fecal microbiota transplantation. (a) Comparisons of the mean relative abundance of gut microbiota at the genera level between the LN mice and HN mice. Genera with relative abundance above 1% are shown in the bar plot. “Others” indicates the sum of the bacteria with a relative abundance less than 1%. (b) Principal coordinate analysis (PCoA1 and PCoA2) of the gnotobiotic mice gut microbiota based on Bray-Curtis distance, with community structure differences tested by adonis analysis of variance with 999 permutations. (c) Boxplots of alpha diversity evaluated by Simpson and Pielou indexes between LN and HN mice (***p* < 0.05, Mann-Whitney U test). (d) Discriminative bacteria between LN mice and HN mice. Genera with relative abundance above 1% are included in the analysis. LN mice: mice colonized with the feces from the infant in LN group; HN mice: mice colonized with the feces from the infant in HN group.

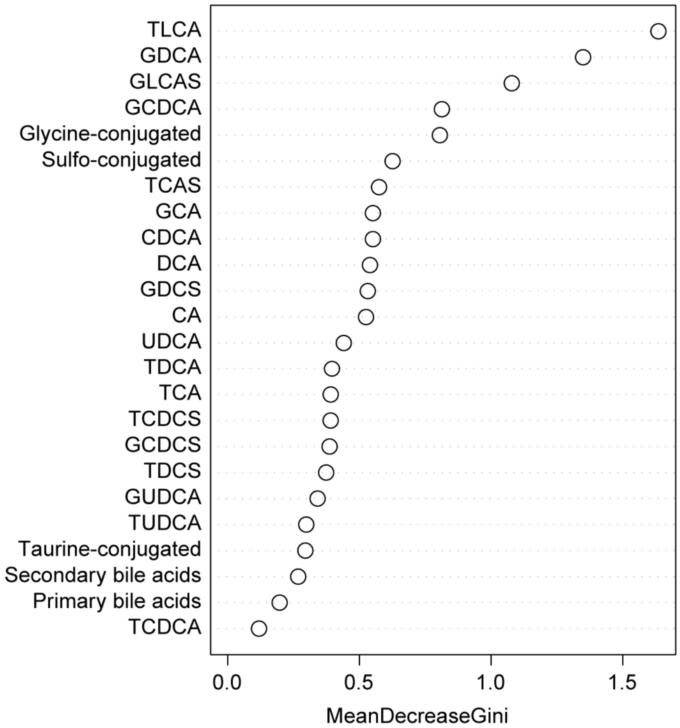

Figure S4. Important bile acids from Random Forests for classifying low obesity risk and high obesity risk infants in the Zhengzhou cohort.

Supplementary Tables

Table S1. Clinical information of the donors for fecal microbiota transplantation.

	**LN Donor**	**HN Donor**
Infant age at feces sampling (days)	9	10
Infant age at breast milk sampling (days)	10	10
Gestational age in days	268	273
Infant sex	Female	Male
Delivery mode	Natural delivery	Natural delivery
Feeding pattern at sampling	Mostly breastmilk feeding	Mostly breastmilk feeding
Breast milk Neu5Ac concentration (μg/mL)	9.74	20.06

LN: Low Neu5Ac group; HN: High Neu5Ac group.

Table S2. Fold change of fecal bile acids between LN and HN infants.

Category	Conjugated	Bile acids	LN group Mean (SD)	HN group Mean (SD)	Fold change	*p*
Primary	Taurine-/Sulfo-	TCAS	0.77% (1.21%)	1.35% (3.23%)	0.81	0.16
Primary	Glycine-/Sulfo-	GCDCS	2.04% (3.67%)	2.33% (2.39%)	0.19	0.10
Secondary	Unconjugated	UDCA	0.50% (1.75%)	0.18% (0.66%)	-1.48	0.12
Secondary	Taurine-/Sulfo-	TDCS	0.09% (0.29%)	0.71% (1.90%)	3.00	0.04
Secondary	Glycine-	GUDCA	0.03% (0.06%)	0.03% (0.04%)	0.03	0.21
Primary	Glycine-	GCA	5.12% (12.84%)	5.84% (10.10%)	0.19	0.05
Secondary	Taurine-	TUDCA	0.03% (0.03%)	0.02% (0.02%)	-0.43	0.73
Primary	Taurine-	TCA	4.98% (10.45%)	10.63% (13.89%)	1.09	0.09
Primary	Unconjugated	CDCA	9.33% (8.35%)	6.41% (8.69%)	-0.54	0.06
Primary	Glycine-	GCDCA	1.15% (2.27%)	3.12% (8.69%)	1.45	0.13
Second	Unconjugated	DCA	0.06% (0.10%)	0.05% (0.16%)	-0.12	0.23
Primary	Taurine-	TCDCA	1.46% (2.89%)	7.25% (9.79%)	2.31	0.03
Secondary	Glycine-	GDCA	0.00% (0.01%)	0.01% (0.03%)	0.98	0.03
Secondary	Taurine-	TDCA	0.01% (0.05%)	0.02% (0.04%)	0.34	0.43
Secondary	Taurine-	TLCA	0.00% (0.00%)	0.00% (0.00%)	0.61	0.05
Primary	Unconjugated	CA	66.16% (31.91%)	44.83% (40.31%)	-0.56	0.16
Secondary	Glycine-/Sulfo-	GDCS	0.16% (0.57%)	1.08% (3.16%)	2.79	0.09
Secondary	Glycine-/Sulfo-	GLCAS	0.16% (0.43%)	0.76% (1.30%)	2.28	0.03
Primary	Taurine-/Sulfo-	TCDCS	7.96% (15.53%)	15.39% (16.80%)	0.95	0.02
		Primary	98.97% (2.16%)	97.15% (6.14%)	-0.03	0.16
		Secondary	1.03% (2.16%)	2.85% (6.14%)	1.47	0.17
		Glycine-conjugated	8.65% (17.39%)	13.16% (17.70%)	0.61	0.10
		Taurine-conjugated	15.31% (24.29%)	35.37% (30.53%)	1.21	0.02
		Sulfo-conjugated	11.18% (19.43%)	21.61% (22.79%)	0.95	0.02

Primary indicates the sum of TCAS, GCDCS, GCA, TCA, CDCA, GCDCA, TCDCA, CA and TCDCS. Second indicates the sum of GDCS, UDCA, TDCS, GLCAS, DCA, GUDCA, TDCA, TUDCA, GDCA and TLCA. Glycine-conjugated bile acids indicate the sum of GDCA, GCDCA, GCDCS, GUDCA, GCA, GDCS and GLCAS. Taurine-conjugated bile acids represent the sum of TCDCS, TCA, TCDCA, TDCS, TCAS, TDCA, TUDCA and TLCA. Sulfo-conjugated bile acids indicate the sum of TCDCS, GDCS, GCDCS, TDCS, GLCAS and TCAS. Fold changes were calculated as the ratio of each bile acid between the HN group and LN group and converted logarithmically. The significant value was calculated by Mann-Whitney U test.

Table S3. Fold change of cecal bile acids between LN and HN mice.

Category	Conjugated	Bile acids	LN mice Mean (SD)	HN mice Mean (SD)	Fold change	*p*
Primary	Taurine-/Sulfo-	TCAS	94.60% (4.51%)	95.02% (2.85%)	0.01	1.000
Primary	Glycine-/Sulfo-	GCDCS	0.01% (0.01%)	0.01% (0.00%)	-0.66	0.418
Primary	Taurine-/Sulfo-	TCDCS	0.28% (0.08%)	0.85% (0.41%)	1.58	0.016
Primary	Glycine-	GCA	0.01% (0.01%)	0.01% (0.00%)	-1.29	0.089
Primary	Taurine-	TCA	2.98% (2.37%)	3.37% (2.31%)	0.18	0.631
Primary	Glycine-	GCDCA	0.10% (0.05%)	0.09% (0.02%)	-0.12	1.000
Primary	Taurine-	TCDCA	0.04% (0.01%)	0.04% (0.01%)	0.05	0.872
Primary	Unconjugated	CA	1.50% (1.78%)	0.07% (0.05%)	-4.51	0.004
Primary	Unconjugated	CDCA	0.05% (0.03%)	0.03% (0.02%)	-0.55	0.574
Secondary	Unconjugated	LCA	0.00% (0.00%)	0.00% (0.00%)	-0.13	0.484
Primary	Unconjugated	UDCA	0.21% (0.19%)	0.19% (0.16%)	-0.15	0.522
Secondary	Taurine-/Sulfo-	TDCS	0.04% (0.02%)	0.12% (0.04%)	1.54	0.006
Primary	Glycine-	GUDCA	0.00% (0.00%)	0.00% (0.00%)	-0.31	0.715
Primary	Taurine-	TUDCA	0.17% (0.09%)	0.20% (0.09%)	0.24	0.522
–	–	Primary bile acids	99.96% (0.02%)	99.88% (0.04%)	0.00	0.006
–	–	Secondary bile acids	0.04% (0.02%)	0.12% (0.04%)	1.51	0.006
–	–	Glycine-conjugated	0.12% (0.06%)	0.10% (0.03%)	-0.25	0.749
–	–	Taurine-conjugated	98.11% (2.01%)	99.61% (0.23%)	0.02	0.055
–	–	Sulfo-conjugated	94.93% (4.43%)	96.00% (2.62%)	0.02	0.873

Primary bile acids represent the sum of TCAS, GCDCS, TCDCS, GCA, TCA, GCDCA, TCDCA, CA, CDCA, UDCA, GUDCA and TUDCA. Secondary bile acids indicate the sum of LCA and TDCS. Glycine-conjugated bile acids indicate GCDCS, GCA, GCDCA and GUDCA. Taurine-conjugated bile acids represent the sum of TCAS, TCDCS, TCA, TCDCA, TDCS and TUDCA. Sulfo-conjugated bile acids bile acids represent the sum of TCAS, GCDCS, TCDCS and TDCS. Fold changes were calculated as the ratio of each bile acid between the HN mice and LN mice and converted logarithmically. The significant value was calculated by Mann-Whitney U test.

Table S4. Fold change of cecal bile acid conversion between LN and HN mice.

**Bile acid conversion**	**LN mice Mean (SD)**	**HN mice Mean (SD)**	**Fold change**	***p***
CA/CDCA	29.40 (28.06)	1.79 (0.95)	-4.03	0.004
GCA/CA	0.01 (0.00)	0.14 (0.12)	3.66	0.004
GCDCA/CDCA	2.53 (0.97)	3.24 (1.39)	0.36	0.522
TCA/CA	2.75 (0.92)	63.46 (24.81)	4.53	0.004
TCDCA/CDCA	1.00 (0.33)	1.43 (0.51)	0.52	0.200
TCAS/TCA	51.58 (32.91)	41.82 (25.25)	-0.30	0.631
TCDCS/TCDCA	8.08 (3.75)	21.15 (9.53)	1.39	0.010
GCA/TCA	0.00 (0.00)	0.00 (0.00)	-1.01	0.010
GCDCA/TCDCA	2.52 (0.80)	2.25 (0.35)	-0.17	0.522
TCDCA/TUDCA	0.25 (0.05)	0.23 (0.07)	-0.14	0.522
GCDCA/GUDCA	130.64 (48.49)	145.72 (52.08)	0.16	0.873
Deconjugated_ratio	0.02 (0.02)	0.00 (0.00)	-2.60	0.037
G_con/unconjugated	2.54 (0.97)	3.38 (1.50)	0.42	0.522
T_con/unconjugated	3.75 (1.20)	64.89 (25.09)	4.11	0.004
S_con/unconjugated	59.66 (34.40)	62.97 (25.32)	0.08	0.631
G_con/T_con	0.00 (0.00)	0.00 (0.00)	-0.28	0.749

Deconjugate ratio means the sum of CA, CDCA, LCA and UDCA divided by the sum of all the cecal bile acids. G_con/unconjugated bile acids indicate the sum of GCA/CA and GCDCA/CDCA. T_con/unconjugated bile acids indicate the sum of TCA/CA and TCDCA/CDCA. S_con/unconjugated bile acids indicate the sum of TCAS/TCA and TCDCS/TCDCA. G_con/T_con bile acids indicate the sum of GCDCS, TCDCS, GCDCA and GUDCA divided by the sum of TCAS, TCDCS, TCA, TCDCA, TDCS and TUDCA. Fold changes were calculated as the ratio of each bile acid between the HN mice and LN mice and converted logarithmically. The significant value was calculated by Mann-Whitney U test.
